# Pathogenic potential and growth kinetics of Muko virus in mice and human-derived cells

**DOI:** 10.1186/s41182-016-0032-7

**Published:** 2016-10-11

**Authors:** Gianne Eduard L. Ulanday, Satoshi Shimada, Ngwe Tun Mya Myat, Takeshi Nabeshima, Kouichi Morita, Daisuke Hayasaka

**Affiliations:** 1 Department of Virology, Institute of Tropical Medicine (NEKKEN), 1-12-4 Sakamoto, Nagasaki, 852-8523 Japan; 2 Leading Graduate School Program, Nagasaki University, 1-12-4 Sakamoto, Nagasaki, 852-8523 Japan

**Keywords:** Muko virus, Tick-borne virus, Mouse model, Human-derived cells

## Abstract

**Background:**

Ticks have been long known as vectors of various pathogens, some of which can cause high fatality rates among infected individuals. Our enhanced tick surveillance around Nagasaki, Japan, led to the isolation and identification of a new strain of a recently identified *Orbivirus*, Muko virus (MUV). The orbiviruses have a wide host range, including humans, and is related to a spectrum of clinical outcomes. However, the zoonotic potential of some members of the genus, although reported, were not clearly elucidated. Hence, it is imperative to characterize newly isolated orbiviruses and investigate its ability to endanger public health.

**Methods:**

In this study, we explored the in vivo pathogenicity of a newly isolated MUV strain (MUV-Hay) using a mouse model and demonstrated its growth kinetics in human-derived cells.

**Results:**

Our results showed the ability of MUV-Hay to propagate in human neuronal and renal cells with some cytopathic effect. Furthermore, intracerebral inoculation of our new isolate caused high mortality in adult A129 mice.

**Conclusion:**

Our study provided a first step to experimentally test the hypothesis, that MUV can replicate and produce cytopathic effect in human cells and demonstrate virulence in adult mice.

## Background

Ixodid ticks transmit a number of viruses, including those of the families *Bunyaviridae*, *Flaviviridae*, *Reoviridae*, *Rhabdoviridae*, and *Orthomyxoviridae* [1–3]. Tick-borne viruses include highly pathogenic agents such as the tick-borne encephalitis virus (TBEV), Crimean-Congo hemorrhagic fever virus (CCHFV), and severe fever with thrombocytopenia syndrome virus (SFTSV).

Of those mentioned, cases of tick-borne encephalitis (TBE) and severe fever with thrombocytopenia syndrome (SFTS) have been reported in Japan [4, 5]. A TBE case was confirmed in the southern area of Hokkaido, north island of Japan, and TBEV was isolated from *Ixodes ovatus* in the endemic area [6]. On the other hand, more than 170 cases of SFTS have been identified in western Japan since 2005 (http://kanpoken.pref.yamaguchi.lg.jp/jyoho/page9/sfts_1.php). Although SFTSV has been detected in several species of ticks in China and Korea [7–13], we and another group were not able to isolate SFTSV from ticks collected by flagging during an epidemiological survey [14, 15].

To aid in the planning of public health measures such as early detection of cases and discrimination of viral from bacterial tick-borne infections, field studies are necessary in providing tick infection rates, species distribution, and level of endemicity. Our field collection of ticks aimed to identify viruses that are possible mammalian pathogens and obtain other data as part of tick surveillance in Japan.

Our efforts in the field sampling of ticks resulted to the identification of a new tick-borne virus, Tofla virus (TFLV), belonging to the genus *Nairovirus*, family *Bunyaviridae*, from *Haemaphysalis flava* and *Haemaphysalis formsensis* ticks [16]. Although the infectivity and pathogenicity of TFLV in humans and animals remain unclear, the virus exhibited ability to propagate in both monkey and human-derived cultured cells. TFLV also produced lethal infection in interferon-α/β receptor knockout (IFNAR KO) mice with marked gastrointestinal pathology [16].

Our enhanced tick surveillance in Nagasaki, located on the Japanese island of Kyushu, isolated an infectious agent that produced fatal infection in IFNAR KO mice. Next-generation sequencing (NGS) identified the pathogen as a new strain of *Muko virus* (MUV) which was first reported by Ejiri et al. [17].

The recently identified virus, MUV, belongs to the genus *Orbivirus* of the family *Reoviridae*, initially isolated from *Ixodes turdus* collected in Hyogo within the Kansai region on the Honshu island of Japan. In the same study, they showed that their isolate (MUV-S1) could replicate and induce cytopathic effect in animal-derived cell lines such as BHK-21 (Syrian hamster kidney), Vero E6 (African green monkey kidney), and CCL-141 (duck embryo) and exhibit lethal infection in suckling mice after intracerebral inoculation. In addition, the genetic association of MUV to *Kemerovo* and *Tribec viruses* suggests that MUV may negatively impact human health and be a potential zoonotic agent. Collectively, their results raise the possibility that MUV may also cause disease among other animals including humans [17].

Since the prevention and control of pathogenic orbiviruses depend on current information, critical gaps in knowledge need to be addressed. The identification of host range susceptible to specific *Orbivirus* species and the molecular determinants involved in host specificity needs to be understood [18]. However, the pathogenicity in adult animals and infectivity in human cells were not elucidated in the previous study. Our study, therefore, has focused on in vitro infectivity of MUV in cells of human origin and demonstrates its in vivo virulence among adult mice.

## Methods

### MUV isolation from ticks

**Fig. 1:**
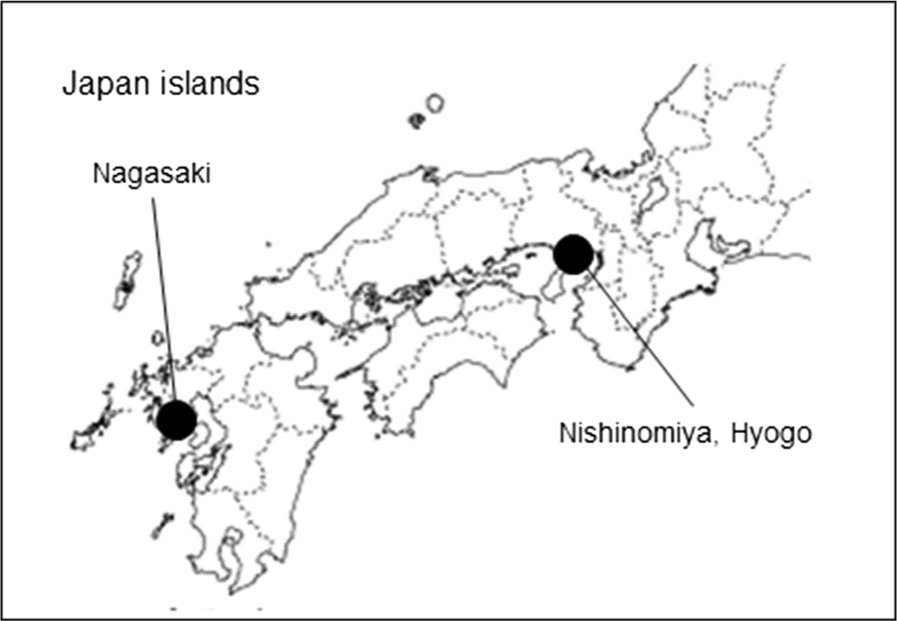
Map showing the location of Nagasaki and Nishinomiya in Japan

Ticks were collected by flagging in Nagasaki in January 2015 (Fig. 1), following the techniques applied in previous studies [14–16]. The pooled *I. turdus* ticks comprising five nymphs were homogenized using Micro Smash™ MS-100R (TOMY DIGITAL BIOLOGY CO., LTD, Tokyo, Japan) with one stainless bead (4.8 Ø) and 0.5 ml of 2 % fetal bovine serum (FBS) in Eagle’s minimum essential medium (EMEM; Nissui Pharmaceutical Co., Tokyo, Japan) per reaction tube at 4500 rpm for 15 s at 4 °C. A total of 100 μl of the supernatant was intraperitoneally inoculated into adult A129 mouse. The post-mortem mouse spleen was collected after 6 days and homogenized in 2 % FBS EMEM.

### MUV genome sequence

RNA was extracted from the homogenized mouse spleen using RNeasy Lipid Tissue Mini Kit (Qiagen, Hilden, Germany). Next-generation sequencing (NGS) was performed using GS Junior 454 (Roche Diagnostic Cooperation, Branford, CT, USA). The full-length sequences were determined using the primers designed from the sequence previously reported [17].

### Phylogenetic analysis

Phylogenetic analyses of the VP region of selected tick-borne *Orbivirus* proteins and amino acid sequences were aligned using the DIALIGN-TX v1.0.2 and TrimAl v1.2 [19–21]. The substitution models were determined by ProtTest v3.4.1 while the phylogenetic trees were reconstructed by the maximum likelihood model using PhyML v3.0.1 [22, 23]. The following accession codes were obtained for phylogenetic analyses from GenBank: Baku virus (AHW57716.1), Colorado tick fever virus (NP_690891.1), Great Island virus (YP_003896058.1), Kemerovo virus (AGG68141.1), Lipovnik virus (ADM88603.1), Muko virus (BAT21343.1), Okhotskiy virus (AHL27158.1), Tribec virus (ADZ96219.1), and Wad Medani virus (YP_009158877.1).

### Virus and cells

The homogenized spleen sample of mouse that was previously inoculated with tick homogenate was inoculated into BHK-21 cells. The infectious culture fluid was harvested 6 days post-inoculation, aliquoted, and kept as master virus stocks.

BHK-21, T98-G (human glioblastoma multiforme), SK-N-SH (human neuroblastoma), and HEK-293 (human embryonic kidney) cells were maintained in EMEM containing 10 % FBS.

### Mice

The IFNAR KO (A129) mice were purchased from B & K Universal Limited and were mated in the facility at Nagasaki University. The B6 mice were purchased from CLEA Japan, Inc. B6 was chosen since it is known to be a standard mouse model in infection research.

### Infection of mice with MUV

Adult (>8 weeks old) A129 mice were assigned into one of the six dosage groups (*n* = 6 per group) prior to subcutaneous inoculation of 10^−2^ to 10^3^ plaque-forming units (pfu) of MUV in EMEM containing 2 % FBS. On the other hand, adult B6 mice were inoculated with a single dose of MUV at 10^3^ pfu (*n* = 6). The mice were weighed daily and observed for clinical signs of disease for 14 days. Weight and mortality ratios were calculated to demonstrate MUV in vivo pathogenicity.

To assess any difference in viral replication within the different organs, A129 mice were subcutaneously infected with 10^2^ pfu of MUV, with three mice per group sacrificed at 1 and 3 days post-inoculation (pi). The thymus, lungs, spleen, liver, kidneys, small intestine, colon, cecum, brain, and spinal cord were removed following perfusion with cold phosphate-buffered saline (PBS). The small intestine, colon, and cecum were washed with cold PBS to remove fecal matter. Brain tissue was further classified and divided into two fractions, the brain cortex and non-cortex. The tissue segments were immediately submerged in RNAlater (Ambion, CA, USA) and stored at −80 °C until they were used.

Viral replication in the different organs was assessed in A129 mice by quantitation of viral copy numbers using real-time RT-PCR. The total RNA was extracted from the previously prepared tissue segments using an RNeasy Lipid Tissue Mini Kit (Qiagen, Hilden, Germany). The MUV-specific primers and probes were designated based on viral segment 1 using Primer3web version 4.0.0 (http://primer3.ut.ee) [24, 25]. Primers used included forward primer MUV1_0745F20: 5′-GGCCAGCTATTCATGGTTCG-3′ and the reverse primer MUV1_0865R20: 5′-CGTCTCCAGCTCCGATATGT-3′, and the PrimeTime® qPCR probe was MUV1_A_int: 5′-/56-FAM/TTATCTCGG/ZEN/AGGGAGGGGAT/3IABkFQ/-3′ (Integrated DNA Technologies, IA, USA). The real-time RT-PCR reactions were performed using a One Step PrimeScript RT-PCR Kit (Takara Bio, Inc., Tokyo, Japan) detected by 7500 Real-time PCR System (Applied Biosystems, CA, USA). The copy numbers were calculated as ratio of the copy numbers of a standard control.

### Determination of MUV infectious titers

Infectious titers of MUV-Hay were determined using a plaque-forming assay. Confluent BHK-21 cells were inoculated with serial dilutions of MUV culture fluid. The virus was allowed to adsorb for 1 h prior to the addition of an overlay medium (MEM with 1 % methylcellulose (Sigma-Aldrich), 2 % FBS). After an incubation of 4 days at 37 °C with 5 % CO_2_, the overlay was carefully removed and washed with PBS. Fixation of cells was done using 4 % paraformaldehyde in PBS at room temperature for at least 30 min. Crystal violet was used to visualize plaques after staining overnight at room temperature. The viral titers were expressed as plaque-forming unit per milliliter.

### Inoculation of MUV into human-derived cell lines

A primary master stock of MUV was used to inoculate T98-G, SK-N-SH, and HEK-293 cells at multiplicity of infection (MOI) of 0.001 and incubated at 37 °C with 5 % CO_2_. After 1 h adsorption, supernatants were removed and fresh EMEM were added. The experiment was performed in triplicate. The culture fluids were harvested daily for 3 days pi and were kept at −80 °C until analysis. Infectious titers of MUV were determined using a plaque-forming assay.

## Results

### Isolation of MUV

**Fig. 2:**
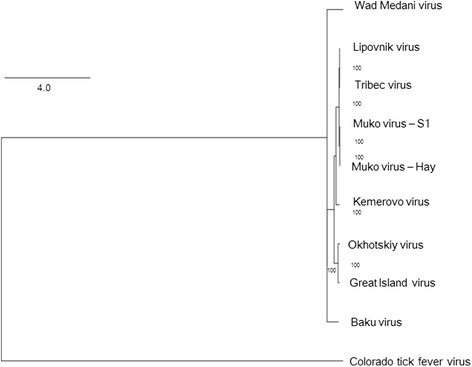
Phylogenetic tree of tick-borne *Orbiviruses*. The phylogenetic tree was constructed based on the VP1 region by LG + G substitution model. Colorado tick fever virus, of the genus *Cortivirus*, was selected as an outgroup. One thousand bootstrap replications were conducted. Bootstrap values (shown as percentage) are described at the nodes

Inoculations with homogenized tick pools of *I. turdus* collected from Nagasaki during the study period resulted in fatal outcomes among A129 mice at 6 days pi. To identify the causative agent of infection, we determined the genome sequence using RNA samples extracted from dead mouse spleen. NGS identified a homologous sequence of MUV, and full sequences of the ten segments were determined by Sanger sequencing. The nucleotide and amino acid sequence identities between the two strains of MUV, MUV-S1 and MUV-Hay, were 96.6–98.7 % and 96.8–99.4 % in each segment, respectively (Table 1). The phylogenetic analyses revealed that the two strains of MUV clustered with Tribec virus and Lipovnik virus within the genus *Oribivirus*, family *Reoviridae* (Fig. 2).

**Table 1:** Amino acid and nucleotide sequence comparison between MUV-S1 and MUV-Hay strains

	Amino acid		Nucleotide	
	Identity (%)	Length (aa)	Identity (%)	Length (bp)
Segment 1	99.1	1284	98.5	3892
Segment 2	99.4	908	98.1	2793
Segment 3	99.0	628	97.3	1935
Segment 4	97.9	529	97.6	1736
Segment 5	98.2	553	97.9	1729
Segment 6	99.4	537	98.7	1668
Segment 7	98.1	368	96.6	1196
Segment 8	98.3	357	97.9	1184
Segment 9	96.8	312	98.0	1034
Segment 10	98.6	214	98.6	705

### Pathogenicity of MUV in adult immunocompetent mice

A previous study has shown that intracerebral inoculation with 10^3^ pfu of MUV-S1 caused fatal infection in suckling mice of outbred ddY mice [17]. However, the pathogenic potential in adult mice is not known. Thus, we subcutaneously inoculated MUV-Hay into adult B6 mice. However, no mice exhibited apparent clinical signs and mortality was not observed (data not shown).

### Pathogenicity of MUV in adult A129 mice

**Fig. 3:**
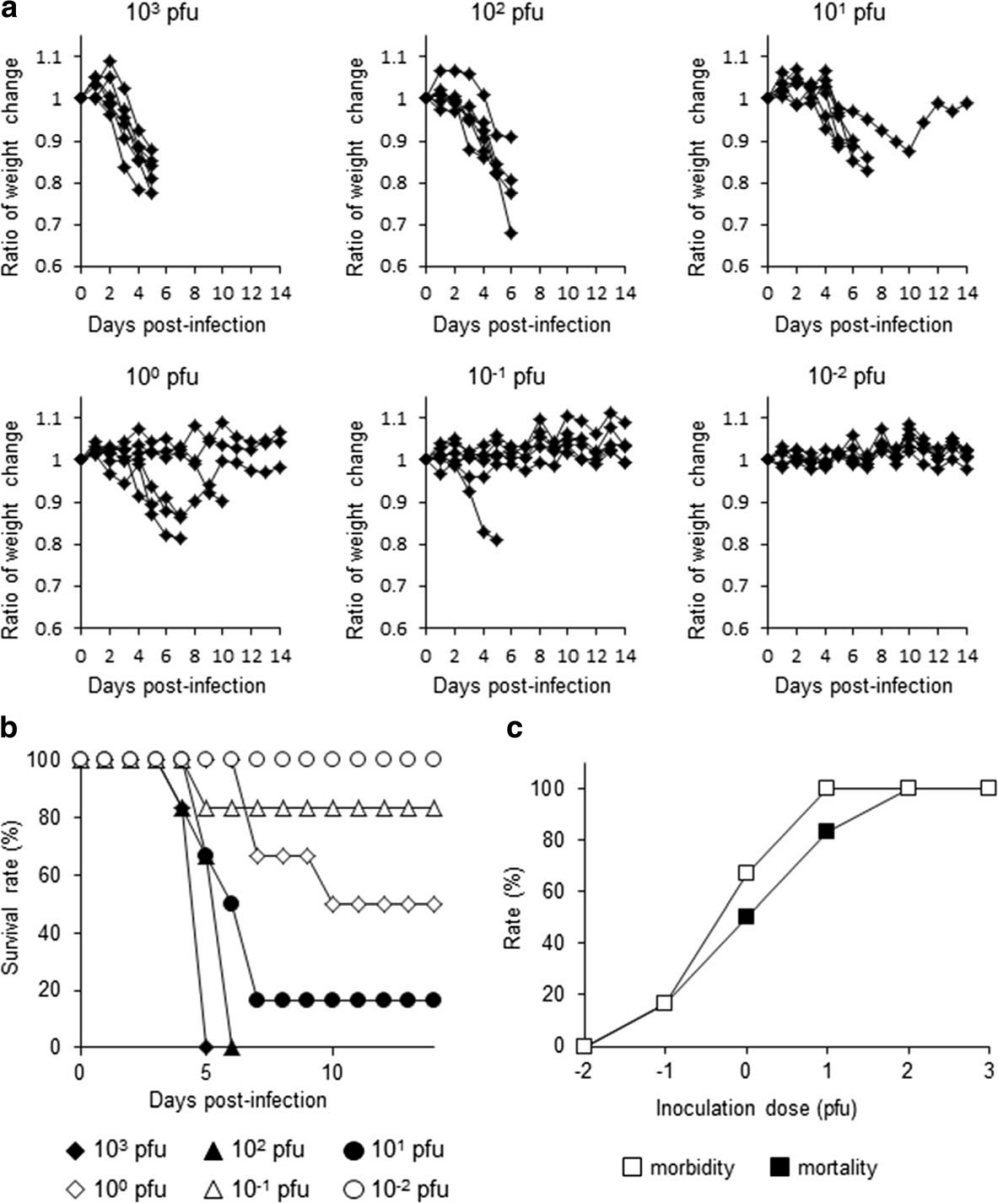
Survival curves of A129 mice in six different dosage groups (total *n* = 36) pertaining to inoculation dose ranging 10^−2^ to 10^3^ pfu of MUV (*n* = 6 per group). Each mouse within the groups was inoculated subcutaneously and observed for 14 days. **a** Weight changes expressed as weight ratios. **b** Survival rate per group. **c** Mortality and morbidity rates. Morbidity of mice was estimated by degree of weight loss

We isolated MUV from dead A129 mice inoculated with a homogenized tick sample. Therefore, we next examined the pathogenicity of the MUV infection in adult A129 mice. Following subcutaneous inoculation with 10^3^ and 10^2^ pfu of MUV-Hay, weight reductions were observed at 2 to 3 days pi and all mice died at 4 to 6 days pi (Fig. 3a, b). Some of the 10^1^ and 10^0^ pfu-inoculated mice recovered after weight reductions (Fig. 3a, c). Inoculation dose-dependent mortality was clearly observed (Fig. 3b, c), and the LD_50_ of MUV was 10^0^ pfu.

**Fig. 4:**
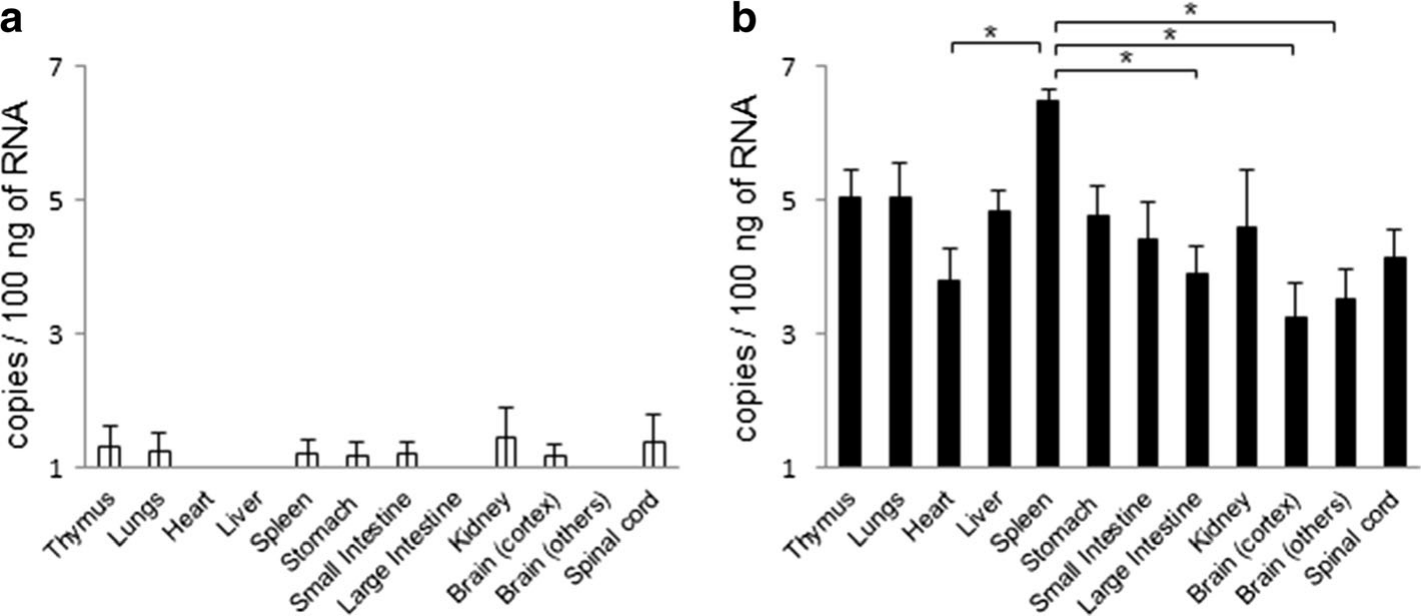
Viral RNA levels in tissues of MUV-infected A129 mice at 1 (**a**) and 3 (**b**) days pi. *Asterisks* show the pairs that exhibit significant differences by Tukey’s multiple comparison test that indicate *P* < 0.05 by the analysis of variance

Viral RNA was detected in some tissues at 1 day pi (Fig. 4a) and increased in every tissue at 3 days pi (Fig. 4b), indicating that MUV rapidly replicated in these tissues. The viral RNA levels varied among the tissues with the levels found in the spleen higher than those of other tissues at 3 days pi (Fig. 4b).

### MUV infectivity and replication in human-derived cell lines

**Fig. 5:**
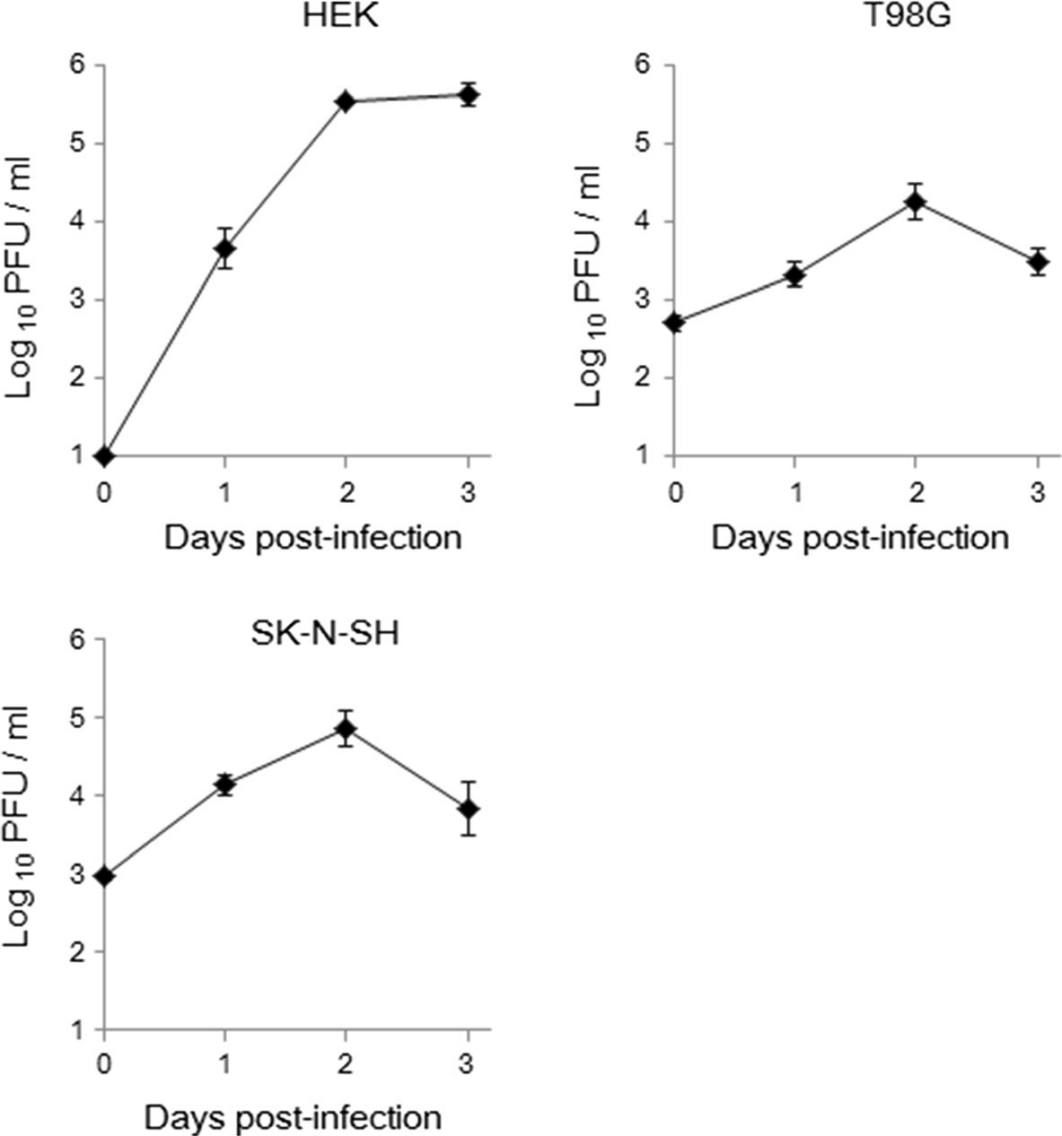
MUV propagation in SK-N-SH, T98-G, and HEK-293 cells. CFs were harvested every 24 h for 3 days pi. Growth curves in the CFs are indicated by plaque-forming unit per milliliter. *Error bars* represent the standard errors

We next examined whether MUV could infect and propagate in human cells. Human-derived SK-N-SH, T98-G, and HEK-293 cells were inoculated with MUV, and infectious virus titers from the different culture fluids were quantified by standard plaque assay. Figure 5 shows the ability of MUV to replicate and propagate in the three selected human cell lines with varied efficiency. The yield of infectious virus at time 0 differed between the neuronal cells (SK-N-SH and T98-G) and the embryonic kidney-derived HEK-293 cells; nevertheless, all three cell lines revealed a time-dependent increase in viral titer. All showed peak growth at day 2 as was reported in other studies [17]. It is notable, however, that the viral titers of neuronal-derived cells decreased after day 2 pi in contrast to those of HEK-293. Cytopathic effect (CPE) of MUV infection on HEK-293 cells was more evident compared to that of T98-G cells while infected SK-N-SH cells showed inconclusive CPE (data not shown). These results suggest that MUV can infect and propagate in human-derived cells.

## Discussion

This study revealed the potential of MUV of infecting mammals including humans. MUV is a newly identified virus isolated from *I. turdus* in Japan; nonetheless, it is not clear whether MUV can cause any obvious pathology among humans and other animals. The main host of *I. turdus* is birds; thus, they may be the primary host of MUV. In support of that statement, the previous report on MUV showed infection and replication of the virus in avian cell culture. In addition, they also demonstrated MUV infectivity in rodent and primate cells [17]. The results of our study strengthen the hypothesis that MUV can infect and replicate on a wide range of hosts, including humans. The observed difference in infectious virus titers between the neuronal and the embryonic kidney-derived cells at time 0 provides an interesting avenue for further research. It is likely that cell-specific receptors and factors, specifically during the initial stages of viral replication, contributed to this phenomenon.

MUV exhibited lethal infection in A129 mice, although the mechanism of death in the MUV-infected A129 mice was unclear. On the other hand, MUV infection did not show any apparent clinical signs in adult B6 mice. This indicates that IFN-I responses are likely to be important for protection against MUV in mice. IFNAR KO mice such as A129 mice are useful animal models for in vivo infections with tick-borne *Bunyaviruses* such as CCHFV, Hazara virus, and TFLV, even though these viruses do not exhibit apparent pathogenicity in immunocompetent mice [16, 26–28]. Therefore, evaluation of antiviral effect of candidate compounds on in vivo viral replication in IFNAR KO mice can be a solution. Since these mice have minimal interferon-mediated immune response, any observed effect can be attributed to the compound administered. These suggest that A129 mice may be used as an alternative model for testing antiviral agents against tick-borne *Bunyaviruses* as well as *Orbiviruses*.

The *Orbiviruses* have a wide range of hosts, from ruminants to humans and exhibiting varied clinical outcomes. In fact, Bluetongue virus, the prototype *Orbivirus*, has caused devastating outbreaks in livestock leading to high economic losses while another member, Epizootic hemorrhagic disease virus, causes a hemorrhagic disease of deer [18, 29, 30]. The phylogenetic analysis revealed that MUV clustered with Lipovnik virus and Tribec virus and therefore suggests the possibility of MUV to be a zoonotic agent.

MUV-S1 and MUV-Hay were isolated from ticks in Nishinomiya and Nagasaki, respectively [17]. The distance between Nishinomiya and Nagasaki is approximately 550 km. *I. turdus* is distributed over a large area of Japan. Therefore, MUV may be distributed throughout Japan and may naturally circulate within ticks and animals including birds. Thus, further investigations such as seroepidemiological surveys of human and animal samples are an important priority to determine the level of infectivity among humans and other animals. In addition, further in vitro experiments using other cell lines originating from tissues of both human and nonhuman mammals will provide useful information in understanding the infectivity and tissue tropism of MUV.

Although our results do not strongly point out that MUV directly associated with pathogenicity to humans, we also cannot rule out the possibility of MUV to be an important human pathogen. Our work provided a first step to experimentally test the hypothesis that MUV can replicate and produce CPE in human cells, which was not included in the previous study [17].

## Conclusions

We described in this study the growth characteristics of a newly isolated strain of MUV using human cell lines and also demonstrated its pathogenicity using a mouse model. Our isolation of a new MUV strain from ticks at a location far from the initial report warrants further investigation. Seroepidemiological surveys of human and animal samples throughout the region are of importance to determine the level of infectivity among humans and other animals. The possible role of interferon during the course of MUV infection should also be further elucidated. Thus, further in vitro experiments using other cell lines originating from tissues of both human and nonhuman mammals will provide useful information in understanding the infectivity and tissue tropism of MUV.

## Abbreviations

MUV: Muko virus
TBE: Tick-borne encephalitis
TBEV: Tick-borne encephalitis virus
CCHFV: Crimean-Congo hemorrhagic fever virus
SFTS: Severe fever with thrombocytopenia syndrome
SFTSV: Severe fever with thrombocytopenia syndrome virus
TFLV: Tofla virus
IFNAR KO: Interferon-α/β receptor knockout
NGS: Next-generation sequencing
EMEM: Eagle’s minimum essential medium
FBS: Fetal bovine serum
pi: Post-inoculation
pfu: Plaque-forming unit
CPE: Cytopathic effect
